# Intestinal Parasites in a Rural Highland Tourist Community of Nepal: Diversity, Prevalence, and Associated Factors in Humans and Livestock

**DOI:** 10.1002/puh2.70272

**Published:** 2026-05-11

**Authors:** Kishor Pandey, Niten Bharati, Yamini Chhetri, Rachana Bhusal, Madan Nepal, Zainuddin Ansari, Minu Shilpakar, Anju Karmacharya, Shaniya Bhusal, Navin Kumar Yadav, Tsunami Thapa Magar, Muna Bhattarai, Bimala Bhattarai, Arti Neupane, Shristi Bhandari, Jitendra Gautam, Siddha Raj Ojha, Anisha KC, Dharmaraj Patel, Ganesh Chaudhary, Sandhya Gautam, Merina Lama, Janak Raj Subedi, Pitambar Dhakal, Mahendra Maharjan, Rajendra Prasad Parajuli

**Affiliations:** ^1^ Central Department of Zoology Institute of Science and Technology Tribhuvan University Kathmandu Nepal; ^2^ Herbert Wertheim School of Public Health and Human Longevity Science University of California San Diego (UCSD) San Diego California USA

**Keywords:** associated factors, intestinal parasitic infections, livestock, Nepal, zoonosis

## Abstract

**Background:**

In Nepal, the close and longstanding human–livestock relationship, vital for subsistence and livelihoods, also creates opportunities for potential zoonotic exposure to intestinal parasites, which remain a persistent and under‐recognized public health challenge.

**Objective:**

This study investigated the prevalence, associated factors, and potential zoonotic implications of intestinal parasitic infections (IPIs) in Sermathang, located in Nepal's Sindhupalchok District.

**Methods:**

Fecal samples were collected from 122 humans and 37 livestock and analyzed using direct smear methods along with sedimentation and flotation concentration techniques.

**Results:**

Among human participants, 27 (22.1%) tested positive for intestinal parasites, with *Ascaris lumbricoides* being the most frequently detected (9.8%), followed by *Entamoeba histolytica* (4.9%) and hookworms (1.6%). In livestock, 27 samples (72.97%) were found to be infected, with common parasites including *Toxocara* species, *Strongyloides* species, and *Ascaris* species. Although individuals involved in farming, those living in non‐concrete houses, and those who consumed raw or undercooked meat had adjusted odds ratios (aORs) of 1.03 (95% CI: 0.40–2.63), 1.15 (0.42–3.15), and 1.20 (0.28–5.20), respectively, none of these associations reached statistical significance.

**Conclusion:**

Although host‐specific species were observed, parasites belonging to the same genera (*Toxocara*, *Strongyloides*, and *Ascaris*) were detected separately in human and livestock samples from the same households. However, microscopy limitations prevent the assessment of zoonotic transmission, underscoring the need for molecular studies to confirm potential cross‐species links.

## Graphical abstract

This cross‑sectional observational study assessed the prevalence and diversity of intestinal parasitic infections in humans and livestock in a rural highland community of Nepal. Intestinal parasites were detected in 22% of human participants and 73% of livestock, with overlapping parasite genera observed within the same households. The findings highlight potential human–livestock exposure interfaces and reinforce the need for integrated parasite control strategies within a One Health framework.

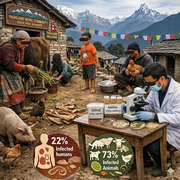

## 1 Introduction

Zoonotic diseases refer to infections that can be transmitted naturally between animals and humans. Zoonotic diseases are responsible for approximately 60% of all known infectious diseases in humans and account for about 75% of emerging infectious diseases globally [1]. A wide variety of pathogens, including viruses, bacteria, fungi, protozoa, and parasites, are capable of causing zoonoses [2]. Several intestinal parasites are important zoonotic agents, posing health risks to both humans and animals, particularly in close human–livestock settings, frequently infecting humans through the fecal–oral route—typically via consumption of food or water contaminated with animal waste [3]. Environments where humans and animals interact closely increase the likelihood of transmission. These include agricultural villages with livestock and homes with domestic pets [4]. Such settings act as transmission bridges between animals and humans. Common parasites that pose zoonotic risks in these contexts include *Ancylostoma* species (hookworms) and *Echinococcus granulosus* (dog tapeworm), as well as protozoa like *Cryptosporidium* and *Giardia* and *Toxocara* species [4, 5, 6].

Intestinal parasitic infections (IPIs) remain widespread on a global scale, particularly in less‐developed regions. According to the World Health Organization, roughly 1.5 billion people, about 24% of the world's population, are infected with intestinal parasites [7]. The majority of this burden comes from soil‐transmitted helminths (STHs) such as roundworms (*Ascaris lumbricoides*), whipworms (*Trichuris trichiura*), and hookworms, which thrive in environments lacking proper sanitation [7]. These infections are most prevalent in tropical and subtropical areas where warm, humid climates favor parasite lifecycles, with sub‐Saharan Africa, parts of Asia, Latin America, and the Caribbean bearing the highest infection rates [3, 7]. Such parasitic diseases impose a huge health and economic burden, from childhood malnutrition and impaired development to lost productivity in afflicted communities. Populations in poverty are disproportionately affected, as associated factors such as inadequate sanitation, unsafe water, and limited health services facilitate the spread of these infections [3, 7]. These global patterns are particularly relevant to Nepal, where underprivileged populations often live in conditions that favor parasite transmission, and agriculture remains the backbone of the economy. Given the shared human–animal–environment interface in endemic settings, this study is grounded in a One Health perspective.

Livestock plays a central role in rural livelihoods and food security, especially in countries such as Nepal, where agriculture is a mainstay of the economy. Many households share close quarters with domestic animals (e.g., cattle, goats, sheep, pigs, and poultry), a situation that can enable zoonotic transfer of parasites and other pathogens. At the same time, parasitic infections take a direct toll on animal health and agricultural productivity. Intestinal parasites in livestock can cause symptoms such as anorexia, diarrhea, anemia, poor growth, and intestinal damage, with the highest mortality seen in young or immunocompromised animals [8, 9]. The prevalence and species diversity of parasites in animal populations fluctuate based on environmental and management factors; for instance, season and climate (temperature and humidity) influence parasite survival and transmission, different geographic areas harbor varying parasite fauna, and the quality/frequency of veterinary care (including deworming) along with whether animals are stray or confined can all affect infection rates [8, 10, 11, 12, 13]. In small ruminants (sheep and goats), which are important for Nepalese farmers, uncontrolled IPIs are one of the most serious constraints on productivity [14]. These parasites cause economic losses by reducing meat/milk yields, causing animal deaths, and necessitating costly treatment and control measures [14]. The challenge is amplified in tropical regions where year‐round favorable conditions allow heavy parasite burdens to persist on pastures [7, 15]. Antiparasitic drugs are widely used for control, but issues like anthelmintic resistance are emerging [14], underscoring the need for integrated parasite management.

Zoonotic parasites pose serious health risks to both humans and animals. However, there is still a scarcity of comprehensive data on their distribution among Nepal's domestic, stray, and farm animal populations [12, 16]. The lack of data is concerning, as animals living alongside humans or entering the food chain can carry high parasite loads, posing serious public health risks. This study aimed to quantify the prevalence and species diversity of IPIs in humans and domestic animals in Nepal and to explore the co‐occurrence of parasite genera in humans and domestic animals and to assess their potential zoonotic relevance between people and their animals. By mapping these overlaps, the study seeks to generate baseline evidence for integrated control strategies under the One Health framework.

## 2 Materials and Methods

### 2.1 Study Area

**FIGURE 1:**
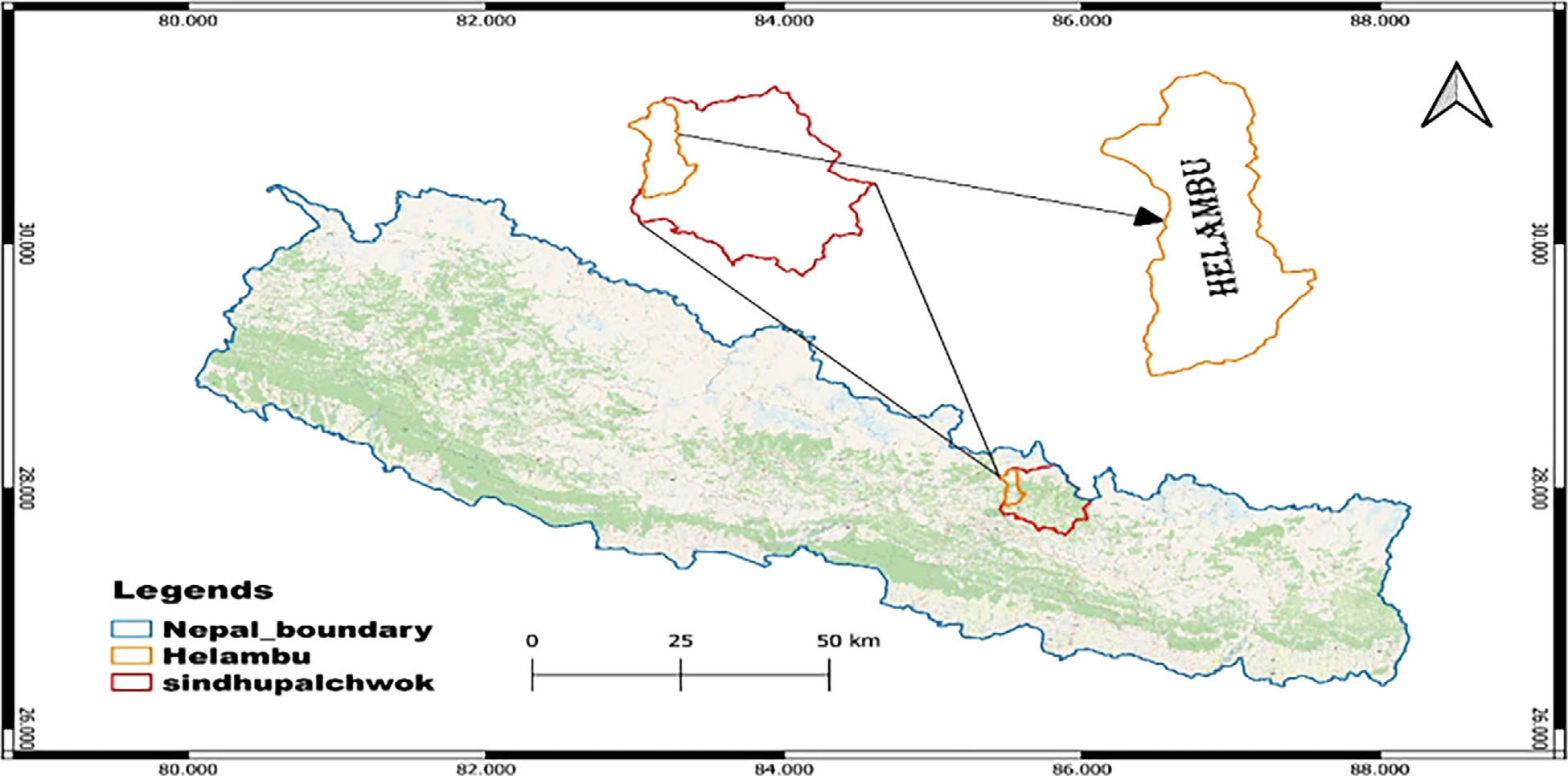
Map of Nepal showing the study area (Helambu Rural Municipality).

Helambu Rural Municipality, situated in Nepal's central Bagmati Province within the Sindhupalchok district, is known for its scenic beauty and cultural richness (Figure 1). Spanning an area of about 287.6 km^2^, it lies at the geographic coordinates 28.05° N and 85.53° E. According to the National Statistics Office (2021), it has a population of 17,497, comprising a diverse mix of ethnic communities such as the Hyolmo, Sanyayi, Sherpa, Gurung, Gharti, Brahmin, Chhetri, Tamang, Newar, Magar, and others [17]. The region offers stunning views of the Jugal Himal Range and includes culturally significant locations like Sermathang, Tarkeghyang, Melamchi Khyang, and Ghopteghyang [18]. Helambu is rich in heritage, marked by the presence of monasteries, temples, and manes that reflect its spiritual and cultural depth. Its natural landscapes, welcoming communities, and cultural landmarks make it a favored spot for tourists and trekkers alike.

### 2.2 Sample Collection

Field visits were conducted at the designated study locations, where assigned teams (i.e., students co‐authors) were responsible for collecting the necessary samples. Each individual participant was approached and informed about the purpose and procedures of the study, with verbal consent obtained after fully explaining their rights, including the option to withdraw at any stage. Verbal consent was used due to variable literacy levels and was documented by the field team through participant logs and unique sample identification codes. Participants were carefully instructed on how to collect their own fecal samples using a sterile vial and a clean stick, ensuring that a thumb‐sized portion was taken from the beginning, middle, and end of the first morning stool while avoiding contamination from urine or soil. To prevent any cross‐contamination, fecal samples from cattle were collected directly by the research team. All collected specimens were properly labeled with unique research codes, preserved in 2.5% potassium dichromate solution to safeguard the integrity of any parasites present, and transported to the laboratory at the Central Department of Zoology, Tribhuvan University, for detailed analysis.

### 2.3 Microscopic Examination

All required laboratory materials were prepared in advance and brought to the analysis area. Each collected sample was then examined using standard diagnostic procedures, including direct smear and concentration techniques. Parasite identification was based exclusively on classical morphological criteria using light microscopy. For human samples, parasites were identified to species level when diagnostic features—such as egg or cyst size, shape, wall structure, nuclear characteristics, and staining patterns—allowed confident differentiation; otherwise, identification was restricted to higher taxonomic levels (i.e., hookworms). For animal samples, parasites were conservatively reported at the genus level due to morphological overlap among species and the known limitations of microscopy for definitive species discrimination in livestock parasites.

All slides were initially examined by MSc‐level students specializing in parasitology as part of supervised field training. Samples with uncertain or uncommon morphological features were reviewed by experienced laboratory technicians, and selected positive samples were further verified by senior faculty members with extensive experience in parasitological diagnostics. Duplicate slides were not prepared routinely; however, expert confirmation served as a quality‐control measure to minimize misclassification.

### 2.4 The Direct Smear Method

The direct smear technique was employed to detect helminth eggs, larvae, and protozoan forms such as cysts, oocysts, and trophozoites, using both stained and unstained slide preparations. For the unstained method, a drop of normal saline was placed on a clean microscope slide and mixed with 1–2 drops of the fecal specimen to create a thin, even layer suitable for microscopic observation. In the staining method, a drop of diluted Lugol's iodine was added to a slide and combined with a small amount of the stool sample. Both preparations were examined under a microscope at magnifications of 10× and 40× to identify parasitic structures, following the procedures outlined in previous studies [19, 20, 21].

### 2.5 Concentration Technique

Along with the direct smears, an indirect examination was used for each sample. The concentration techniques were based on two further procedures, that is, sedimentation and flotation.

#### 2.5.1 Flotation Technique

To prepare the sample suspension, roughly 3 g of stool were mixed with 15 mL of saturated sodium chloride solution and then filtered through a tea strainer. A portion of the filtrate (1–2 mL) was transferred into a centrifuge tube containing 13 mL of NaCl solution and centrifuged at 1000 rpm for 15 min. Additional saturated NaCll was added until a convex meniscus formed at the top of the tube, followed by the addition of a drop of methylene blue. A coverslip was carefully placed over the tube and left undisturbed for 30 min. Afterward, the coverslip was removed and examined under a microscope to identify the parasitic stage, following standard diagnostic procedures [22].

#### 2.5.2 Sedimentation Technique

**FIGURE 2:**
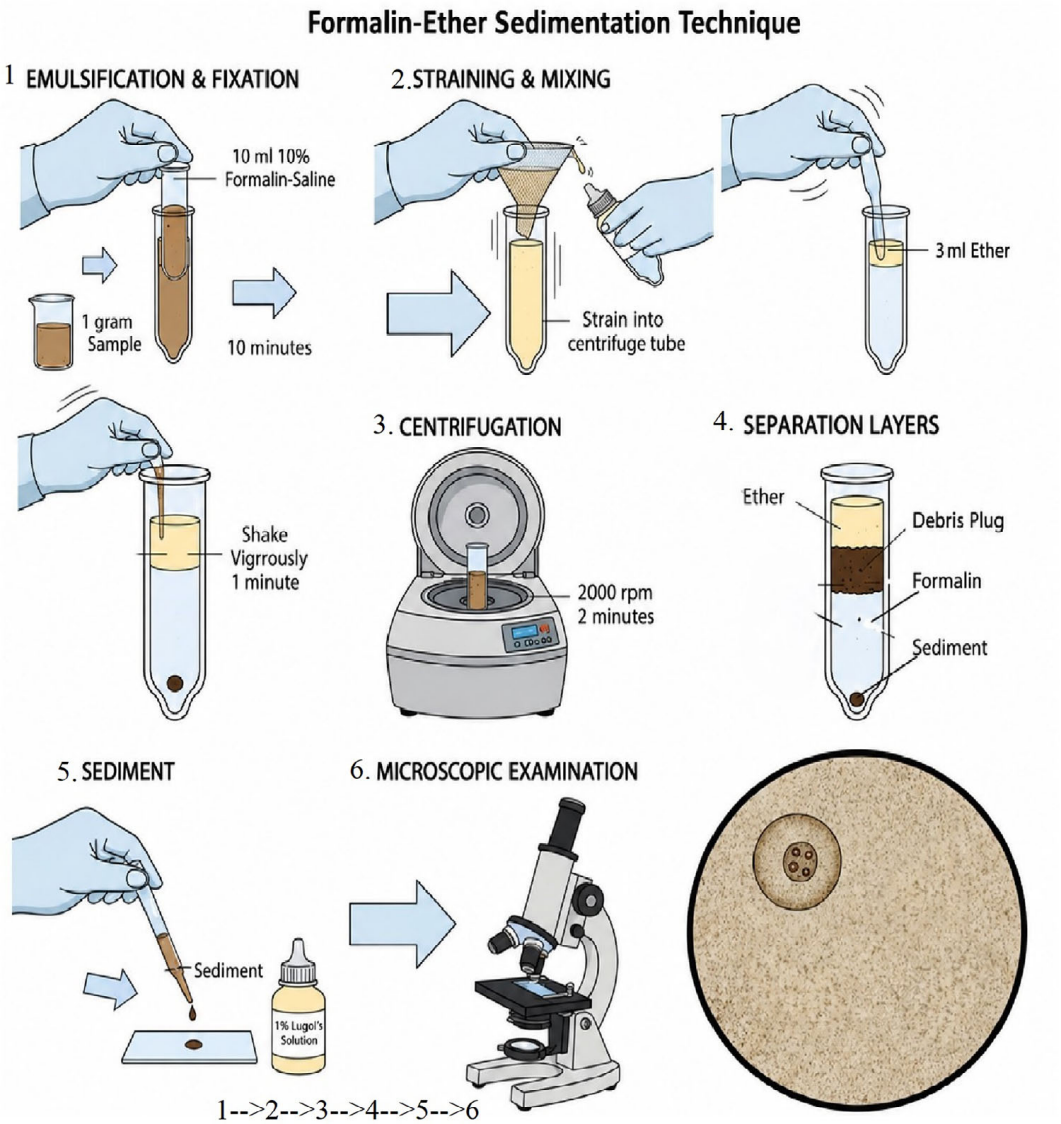
Formalin–ether sedimentation technique.

Fecal sample of 1 g was emulsified in 10 mL of 10% formal‐saline and left for 10 min for fixation. The suspension was strained into a centrifuge tube, mixed with 3 mL of ether, and shaken vigorously for 1 min. After centrifugation at 2000 rpm for 2 min, the layers formed included ether, debris, formalin, and sediment. The sediment was placed on a clean slide with a drop of 1% Lugol's iodine solution, covered with a coverslip, and examined microscopically (Figure 2) [22].

### 2.6 Questionnaire Survey

A structured questionnaire was developed based on insights from previous studies [19, 20, 21, 23, 24] to assess potential factors associated with IPIs. The questionnaire captured information on sociodemographic, socioeconomic, hygiene, lifestyle, and animal‐related factors relevant to parasite transmission. To ensure clarity and avoid redundancy, the questionnaire was carefully designed and administered in the local language, with an English‐translated version provided as Supporting Information. Household size was categorized as ≤4 versus >4 members based on the sample distribution and local household norms. Percentages for selected questionnaire items were calculated based on valid responses only, as participants were not required to answer all questions and non‐responses occurred for some dietary variables.

### 2.7 Data Analysis

The data were summarized using means and standard deviations for continuous variables and frequencies and percentages for categorical variables. Characteristics and prevalence of IPIs were compared between participants with and without IPIs. Group differences were evaluated using independent *t*‐tests for continuous variables and chi‐square tests or Fisher's exact tests for categorical variables, as appropriate.

Associations between selected sociodemographic, lifestyle, and contextual factors and IPIs among humans were examined using bivariate logistic regression, followed by exploratory multivariable logistic regression. Variables with odds ratios (ORs) >1.0 in bivariate analyses were entered into the multivariable model for mutual adjustment, acknowledging the limited sample size and number of outcome events. Correlations among candidate variables were assessed prior to multivariable modeling to minimize multicollinearity. ORs and adjusted odds ratios (aORs) with 95% confidence intervals (CIs) are reported, and statistical significance was defined as *p* <  0.05. All analyses were performed using SPSS statistical software.

## 3 Results

Table 1 summarizes the demographic and socioeconomic characteristics of participants with and without IPIs. The overall mean age was 53.76 years, with infected participants being slightly younger than uninfected individuals (51.81 vs. 54.33 years), although this difference was not statistically significant. Household size was comparable across groups. Although none of the sociodemographic or socioeconomic variables showed statistically significant associations with IPIs, several noteworthy patterns were observed. Participants with IPIs were more likely to report literacy (63.0% vs. 50.0%) and use tap or other improved water sources (63.0% vs. 44.2%). Participants predominantly lived in concrete houses (≈75%) and reported near‐universal access to toilets (≈99%), reflecting relatively good sanitation infrastructure. Agriculture was the main occupation for approximately two‐thirds of participants, suggesting similar occupational exposure profiles. Recent diarrhea was uncommon (4.1%) and evenly distributed. Overall, these findings suggest that IPIs in this population may not be strongly associated with conventional socioeconomic indicators.

**TABLE 1:** Characteristic features of the study participants (*n* = 122).

Characteristics	Without IPIs (*n* = 95)	With IPIs (*n* = 27)	*p* value	Overall (*n* = 122)
Demographic characteristics	Mean (SD)/*n* (%)	Mean (SD)/*n* (%)		Mean (SD)/*n* (%)
Age (in years)	54.33 (23.56)	51.81 (22.77)	0.312^a^	53.76 (23.32)
Numbers of family member in home	3.51 (1.90)	3.65 (2.70)	0.374^a^	3.54 (2.08)
**Socioeconomic (SES) characteristics**				
*Household size?*				
Live ≤4 members in house	72 (75.8)	21 (77.8)	0.830^b^	93 (76.2)
Live >4 members in house	23 (24.2)	6 (22.2)		29 (23.8)
*Can you read and write?*				
Yes	47 (49.5)	17 (63.0)		64 (52.5)
No	48 (50.5)	10 (37.0)	0.234^b^	58 (47.5)
*What is your occupation?*				
Other	32 (33.7)	9 (33.6)	0.973^b^	41 (33.6)
Agriculture	63 (66.3)	18 (66.7)		81 (66.4)
*Source of water?*				
Tap or other	42 (44.2)	17 (63.0)	0.085^b^	59 (48.4)
Well	53 (55.8)	10 (37.0)		63 (51.6)
*Type of house?*				
Concrete	72 (75.8)	20 (74.1)	0.855^b^	92 (75.4)
Mud or others	23 (24.2)	7 (25.9)		30 (24.6)
*Do you have toilet?*				
Yes, we have	95 (100)	26 (96.3)	0.221^c^	121 (99.2)
No, open field defecation	0 (0)	1 (3.7)		1 (0.8)
*Do you have diarrhea within 7 days?*				
No, I do not	91 (95.8)	26 (96.3)	0.694^c^	117 (95.9)
Yes, I do	4 (4.2)	1 (3.7)		5 (4.1)

Abbreviation: IPIs, intestinal parasitic infections.

^a^ Independent *t*‐test.

^b^ Chi‐square test.

^c^ Fisher's exact test where the cell has a count less than 5.

Table 2 presents the behavioral and lifestyle characteristics of the study participants. Overall, there were no significant differences between infected and uninfected individuals in terms of most of these behaviors. A majority of the participants indicated that they use soap when washing their hands, regularly trim their nails, do not eat raw meat, and often wear sandals while outdoors. On the other hand, most of infected participants and overall participants reported not deworming within 3 months, being nonvegetarian, and did not wash vegetables and fruits prior to eating.

**TABLE 2:** Behavioral and lifestyle characteristics of the study participants (*n* = 122).

Characteristics	Without IPIs (*n* = 95)	With IPIs (*n* = 27)	*p* value	Total
Lifestyle and behavioral characteristics	*n* (%)	*n* (%)		*n* (%)
***Do you use soap while washing your hands?***				
Yes	88 (92.6)	27 (100)	0.346^a^	115 (94.3)
No	7 (7.4)	0 (0)		7 (5.7)
***Did you trim nails regularly every week?***				
Yes	52 (54.7)	19 (70.4)	0.146^b^	71 (58.2)
No	43 (45.3)	8 (29.6)		51 (41.8))
***Do you wash vegetables and fruits prior eating?***				
Yes	22 (23.2)	9 (33.3)	0.284^b^	31 (25.4)
No	73 (76.8)	18 (66.7)		91 (74.6)
***Do you walk barefoot while outdoors?***				
No	91 (95.8)	27 (100)	0.575^a^	118 (96.7)
Yes	4 (4.2)	0 (0)		4 (3.3)
***Are you vegetarian?*** ^c^				
Yes	15 (16.0)	5 (18.5)	0.720^a^	20 (16.5)
No	79 (84.0)	22 (81.5)		101 (83.5)
***Do you eat raw meat?*** ^c^				
No	79 (90.8)	24 (88.9)	0.720^a^	103 (90.4)
Yes	8 (9.2)	3 (11.1)		11 (9.6)
***Did you deworm within 3 months?***				
Yes	8 (8.4)	4 (14.8)		12 (9.8)
No	87 (91.6)	23 (85.2)	0.772^a^	110 (90.2)

Abbreviation: IPIs, intestinal parasitic infections.

^a^ Fisher's exact test where the cell has a count less than 5.

^b^ Chi‐square test.

^c^ Percentages are based on valid responses (vegetarian: without IPIs *n* = 94; total *n* = 121; raw meat: without IPIs *n* = 87; total *n* = 114).

About a quarter of the collected samples [27 out of 122 (22.1%)] were positive for one or more species of IPIs (Table 3). Among the seven identified species of parasites, two (*Entamoeba histolytica* and *Giardia lamblia*) were protozoa, and five (*A. lumbricoides*, *T. trichiura*, *Strongyloides stercoralis*, *Toxocara canis*, and hookworms) were helminths. Overall, *A. lumbricoides* had the highest prevalence (9.8%), followed by *E. histolytica* (4.9%) and hookworms (1.6%). There was no statistically significant difference in the overall prevalence of IPIs, or in the prevalence of individual protozoan, helminth, or duplet infections, between males and females (Table 3 ). Overall, 13.1%of the samples presented helminth parasites, whereas 5.7% of the samples presented protozoan parasites. One 90‐year‐old male participant had duplet infections with *T. canis* and *G. lamblia*.

**TABLE 3:** Prevalence of intestinal parasitic infections (IPIs) in the study participants (*n* = 122).

Parasite species	Male (%) *n* = 60	Female (%) *n* = 62	Chi‐square *p* value	Total *n* (%) *n* = 122
**Protozoan parasites**				
*Entamoeba histolytica*	3 (5.0)	3 (4.8)	0.644^a^	6 (4.9)
*Giardia lamblia*	1 (1.7)	0 (0)	0.492^a^	1 (0.8)
**Helminth parasites**				
*Ascaris lumbricoides*	6 (10.0)	6 (9.7)	0.619^b^	12 (9.8)
*Strongyloides stercoralis*	1 (1.7)	0 (0)	0.492^a^	1 (0.8)
*Trichuris trichiura*	0 (0)	1 (1.6)	0.500^a^	1 (0.8)
*Toxocara canis*	1 (1.7)	0 (0)	0.492^a^	1 (0.8)
Hookworms	2 (3.4)	0 (0)	0.496^a^	2 (1.6)
Total infection^c^	14 (23.3)	13 (21.0)	0.753^b^	27 (22.1)
Total protozoan infection^c^	4 (6.7)	3 (4.8)	0.715^a^	7 (5.7)
Total helminth infection^c^	9 (15.0)	7 (11.3)	0.591^b^	16 (13.1)
Duplet infection^c^	1 (1.7)	0 (0)	0.492^a^	1 (0.8)

^a^ Fisher's exact test where the cell has a count less than 5.

^b^ Chi‐square test.

^c^ Species counts represent occurrences; individuals with mixed infections appear in multiple species rows. Summary rows reflect unique individuals.

In parallel, fecal samples from 37 animals—including goats, cows, buffaloes, chickens, dogs, and calves—were examined for parasitic infections. The overall prevalence among the animal population was high, with 27 samples (72.97%) testing positive (Table 4). Of the animals examined, 21 of 26 captive animals, 4 of 6 free‐ranging animals, and 2 of 5 animals with mixed housing status were positive; similarly, 22 of 30 adult animals and 5 of 7 calves tested positive. These findings are presented descriptively due to the small sample size.

**TABLE 4:** Prevalence of intestinal parasites among the animal population.

Characters		Total	Positive
**Status of shed**	Captive	26	21
	Free ranging	6	4
	Mixed (captive and free‐range)^a^	5	2
**Type of animal**	Adult	30	22
	Calf	7	5
**Antihelminthic drugs**	Yes	1	0
	No	36	27

^a^ Animals classified as “mixed” were seasonally managed—kept in sheds during crop‐growing periods (e.g., summer) and allowed to roam freely on farmland after harvest (e.g., winter). Given the small animal sample size, subgroup results are presented as raw counts and should be interpreted descriptively rather than comparatively.

Notably, none of the animals that had been administered anthelmintic drugs tested positive for parasites. Microscopic analysis revealed a range of parasitic species, including *Strongyloides* (eggs and larvae) in goats, *Balantidium sulcata* in cows, *Moniezia* spp., *Eimeria* spp., *Toxocara*, *Ascaris* spp., trematode species in buffaloes, and *Ascaridia* spp. in hens.

Table 5 outlines the relationships between any parasitic infections and related factors. The bivariate analysis showed higher point estimates of ORs among participants who worked in agriculture, lived in non‐concrete houses, and ate raw or undercooked meat than did their reference groups or counterparts. However, none of these associations were statistically significant in either the bivariate analysis or the multivariate model. In the mutually adjusted multivariate model, the estimated ORs remained above 1.0 for agriculture [aOR:  1.03, 95% CI:  0.40–2.63], non‐concrete housing [aOR:  1.15, 95% CI:  0.42–3.15], and raw meat consumption [aOR:  1.20, 95% CI:  0.28–5.20], but all CIs included 1.0, indicating no statistically significant associations.

**TABLE 5:** Prevalence and odds ratio of intestinal parasitic infections (IPIs) with respect to behavioral characteristics using logistic regression analysis (*n* = 122).

Socioeconomic (SES) characteristics	Univariate	Multivariate^a^
	OR (95% CI)	aOR (95% CI)
***Gender***		
Male	Ref	
Female	0.87 (0.37–2.05)	
***Household size?***		
Live ≤4 members in house	Ref	
Live >4 members in house	0.89 (0.32–2.48)	
***Can you read and write?***		
Yes	Ref	
No	0.59 (0.24–1.42)	
***What is your occupation?***		
Other	Ref	Ref
Agriculture	1.02 (0.41–2.51)	1.03 (0.40–2.63)
***Source of water?***		
Tap or other	Ref	
Well	0.47 (0.19–1.12)	
***Type of house?***		
Concrete	Ref	Ref
Mud or others	1.10 (0.41–2.92)	1.15 (0.42–3.15)
***Do you have diarrhea within 7 days?***		
No, I do not	Ref	
Yes, I do	0.88 (0.09–8.17)	
**Behavioral and lifestyle characteristics** ^b^		
***Are you vegetarian?***		
Yes	Ref	
No	0.84 (0.27–2.55)	
***Did you trim nails regularly every week?***		
Yes	Ref	
No	0.51 (0.20–1.28)	
***Do you wash vegetables and fruits prior eating?***		
Yes	Ref	
No	0.60 (0.24–1.53)	
***Do you eat raw meat?***		
No	Ref	Ref
Yes	1.23 (0.30–5.02)	1.20 (0.28–5.20)
***Did you deworm within 3 months?***		
Yes	Ref	
No	0.53 (0.15–1.91)	

**^Abbreviations:^ :**aOR, adjusted odds ratio; CI, 95% confidence interval; OR, odds ratio; ref, reference.

^a^ Model adjusted for variables with univariate OR > 1.0.

^b^ Behavioral and lifestyle characteristics such as using soap for handwashing or using sandals while outdoors was not included in the model due to 100% positivity, which resulted in infinite odds ratios and unstable confidence intervals.

## 4 Discussion

IPI prevalence in our study (22.1%) appears moderate when compared to other Nepalese studies since 2010. Several recent community‐based surveys have reported overall IPI rates in the 20%–30% range, similar to our finding. For instance, Chaudhary and Subedi observed a 28.6% infection rate in the Satar and Chaudhary communities of Jhapa [25]; Dhakal and Subedi found 27.3% among the Meche community in eastern Nepal [26]; Gautam et al. reported 27% in the indigenous Badi community of western Nepal [19]. However, IPI prevalence varies widely across Nepal, with some marginalized groups experiencing substantially higher infection rates. For example, Thapa et al. reported a 31.3% prevalence in the Sarki ethnic group of Baglung [27], and Khadka et al. documented an overall 36.6% IPI rate in two highly disadvantaged communities (about 39.8% in the Chepang and 33.3% in the Musahar people) [28]. An even higher prevalence has been recorded, such as an IPI prevalence of 81% in a Musahar community of Saptari District [29], and an exceptionally high prevalence of 97% was observed among a Chepang population in central Nepal [24]. On the other hand, a few studies have reported much lower IPI frequencies (<20%) in certain populations. For example, Tandan et al. noted 11.3% in residents of Nawalpur District [30].

These disparities in IPI prevalence likely reflect a combination of methodological and contextual differences across studies, including variation in geographic setting (lowland vs. hill regions), sanitation coverage, hygiene practices, study populations, diagnostic methods (direct smear vs. concentration techniques), and sampling strategies (community‐based surveys vs. targeted high‐risk groups) [19]. Despite Nepal's ongoing deworming programs [31, 32], a prevalence of approximately 22% (roughly one‐fifth of participants) in our study indicates that transmission is still a concern in the local context. Field observations by our team noted poor sanitary conditions and unhygienic behaviors among participants, for example, inconsistent use of latrines and soap, infrequent deworming, and inadequate washing of vegetables and fruits, which may explain why IPIs remain relatively high. Although 22.1% is not as alarmingly high, it is still significant given that it represents a sizable minority of people harboring parasites. This underscores the need for targeted interventions (improved sanitation and hygiene education in tandem with ongoing mass deworming) to further reduce the IPI burden in such communities.

Among the identified species, *A. lumbricoides* showed the highest prevalence (9.8%), followed by *Entamoeba* histolytica (4.9%) *and* hookworms (1.6%). The dominance of *A. lumbricoides* aligns with previous findings of ours [21] and also from Khadka et al., who reported its prevalence at 15.6%, followed by *Taenia* sp. (6.3%) and hookworm (4.9%) [28] and others [33, 34].

As observed in Parajuli et al., none of the socioeconomic, sociodemographic, lifestyle, and behavioral characteristics were significantly associated with the prevalence of overall IPIs [20]. This lack of association is likely due to the study's relatively small sample size and low infection prevalence, which limited the statistical power to detect significant effects [23]. Notably, other studies with larger sample sizes have detected significant associations for IPIs; for example, involvement in agriculture, poor hand hygiene (not using soap before meals), and consuming raw or undercooked meat were all linked to higher odds of infection in certain communities [19]. Our observed (but nonsignificant) trends of higher infection odds among participants engaged in agriculture, living in non‐concrete houses, and eating raw meat are consistent in direction with these previously reported associations; however, given the limited sample size and lack of statistical significance, these findings should be interpreted cautiously and viewed as hypothesis‐generating rather than confirmatory.

The high prevalence of parasitic infections in livestock observed in this study underscores a substantial parasitic burden and highlights the need for improved husbandry practices and anthelminthic interventions to reduce transmission within animal populations and potentially to humans. The prevalence rate among captive animals (80.76%) was comparable to the 86% among swine raised on farms in western Nepal [13], 80% prevalence in captive goats in Dolakha [12], 62% among bovine calves in Southern Nepal [11], and 86.9% prevalence reported in dairy cattle in Taiwan [35]. The higher prevalence among captive animals may reflect greater exposure to contaminated fodder, including forage collected from multiple locations. Field observations further revealed poor shed hygiene and suboptimal feeding practices, which likely related to an increased risk of parasitic infection. Free‐ranging animals showed a slightly lower prevalence (66.67%), similar to the 72% prevalence reported in stray cattle [36].

Adult livestock showed slightly higher parasite positivity than calves, which may reflect longer cumulative exposure to contaminated environments and repeated grazing cycles; however, given the small animal sample size and descriptive nature of the analysis, this pattern should be interpreted cautiously.

*Strongyloides* eggs and larvae were detected in goats, which may be attributed to poor pasture management and overgrazing, conditions that favor the accumulation and survival of infective larvae on contaminated land [37]. *Bunostomum sulcatum* was observed in cows, and host susceptibility to infection may be influenced by nutritional status alongside other management and environmental factors, as reported in previous studies [35]. In buffaloes, the detection of *Toxocara* spp.,—a parasite typically found in dogs and cats—suggests potential transmission through contaminated fodder or close contact with infected canines. The presence of trematodes (*Fasciola* spp.) in buffaloes may be explained by exposure to molluscan intermediate hosts in grazing areas or waterlogged fodder. Among hens, the occurrence of *Ascaris* spp. could be attributed to poor sanitation, inadequate veterinary care, and free‐roaming behavior that increases their exposure to contaminated soil and intermediate hosts [38]. However, due to the small sample size and absence of formal statistical testing, these patterns should be interpreted with caution and should not be considered conclusive.

Deworming treatment also appeared to have an influence. The only animal that had received antiparasitic medication tested negative for parasitic infection. Although this single case is insufficient to draw definitive conclusions, it supports existing evidence on the effectiveness of regular deworming in parasite control. These observations, though preliminary, reinforce the need for improved animal husbandry practices, including proper sanitation, routine deworming, and better quality fodder to minimize parasitic transmission in livestock populations.

This study has several limitations that must be acknowledged. First, its cross‐sectional design limits the ability to draw causal inferences. The small sample size and reliance on nonrandom, convenience sampling limit the generalizability of the findings and preclude the use of robust statistical analysis. In particular, the limited livestock sample size restricts meaningful subgroup comparisons, and animal results are therefore presented descriptively rather than inferentially. In addition, the limited number of outcome events relative to candidate predictors violates recommended events‐per‐variable thresholds for multivariable modeling; therefore, regression results should be interpreted as exploratory and hypothesis‐generating rather than inferential. Accordingly, all observed differences represent descriptive, correlational associations rather than evidence of causal relationships. Furthermore, several potential confounders and contextual variables were not measured—for instance, whether the infected animals and their caretakers share environments or behaviors that might facilitate cross‐species transmission. The absence of molecular diagnostic methods also restricted our ability to confirm zoonotic links between human and animal infections, and therefore the findings should be interpreted as descriptive rather than evidence of interspecies transmission. Additionally, reliance on morphology‐based microscopy and genus‐level identification of animal parasites precludes definitive species determination and prevents confirmation of zoonotic transmission in the absence of molecular diagnostics. Future studies with larger, representative samples and molecular tools are needed to more precisely elucidate the epidemiology and zoonotic potential of gastrointestinal parasites in rural Nepal.

## 5 Conclusion

Despite nearly two decades of national deworming efforts, the 22.1% prevalence of intestinal parasites in the human population remains a moderate but concerning burden. In contrast, the very high infection rate in animals (∼73%) poses a potential zoonotic concern, although direct transmission between animals and humans could not be assessed in this study due to the absence of paired human–animal data and molecular confirmation. Socioeconomic proxies such as non‐concrete housing, occupational exposure through farming, irregular deworming, and the practice of eating raw meat may be related to ongoing transmission risks. To effectively reduce the parasitic burden in both humans and animals, health education and behavioral change interventions must complement mass deworming initiatives.

## Author Contributions

**Kishor Pandey**: conceptualization, methodology, supervision, formal analysis, writing – review and editing. **Niten Bharati**: field investigation, data collection, laboratory processing, methodology, writing – original draft. **Yamini Chhetri**: field investigation, data collection, laboratory processing, methodology, writing – original draft. **Rachana Bhusal**: field investigation, data collection, laboratory processing, methodology, writing – original draft. **Madan Nepal**: field investigation, data collection, laboratory processing, methodology, writing – original draft. **Zainuddin Ansari**: field investigation, data collection, laboratory processing, methodology, writing – original draft. **Minu Shilpakar**: field investigation, data collection, laboratory processing, methodology, writing – original draft. **Anju Karmacharya**: field investigation, data collection, laboratory processing, methodology, writing – original draft. **Shaniya Bhusal**: field investigation, data collection, laboratory processing, methodology, writing – original draft. **Navin Kumar Yadav**: field investigation, data collection, laboratory processing, methodology, writing – original draft. **Tsunami Thapa Magar**: field investigation, data collection, laboratory processing, methodology, writing – original draft. **Muna Bhattarai**: field investigation, data collection, laboratory processing, methodology, writing – original draft. **Bimala Bhattarai**: field investigation, data collection, laboratory processing, methodology, writing – original draft. **Arti Neupane**: field investigation, data collection, laboratory processing, methodology, writing – original draft. **Shristi Bhandari**: field investigation, data collection, laboratory processing, methodology, writing – original draft. **Jitendra Gautam**: field investigation, data collection, laboratory processing, methodology, writing – original draft. **Siddha Raj Ojha**: field investigation, data collection, laboratory processing, methodology, writing – original draft. **Anisha K.C**.: field investigation, data collection, laboratory processing, methodology, writing – original draft. **Dharmaraj Patel**: field investigation, data collection, laboratory processing, methodology, writing – original draft. **Ganesh Chaudhary**: field investigation, data collection, laboratory processing, methodology, writing – original draft. **Sandhya Gautam**: field investigation, data collection, laboratory processing, methodology, writing – original draft. **Merina Lama**: field investigation, data collection, laboratory processing, methodology, writing – original draft. **Janak Raj Subedi**: field investigation, data collection, laboratory processing, methodology, writing – original draft. **Pitambar Dhakal**: field investigation, data collection, laboratory processing, methodology, writing – original draft. **Mahendra Maharjan**: field investigation, data collection, laboratory processing, methodology, writing – original draft. **Rajendra Prasad Parajuli**: conceptualization, methodology, formal analysis, visualization, software, writing – review and editing.

## Funding

The authors have nothing to report.

## Ethics Statement

Initially, each eligible participant was approached, and the study's objectives and procedures were explained. If they agreed to participate, they were asked to provide informed consent. In cases involving individuals under the age of 18, consent was obtained from both the minor and a parent or legal guardian. For domestic animals, approval was granted by their respective owners. The study was carried out with guidance and cooperation from local stakeholders, ensuring that no harm was caused to any animals involved. This study was conducted as part of the supervised MSc Parasitology Practicum at the Central Department of Zoology, Tribhuvan University, involving non‑interventional educational field activities. As the study did not involve clinical interventions, invasive procedures, or collection of identifiable personal data, formal institutional ethics committee approval was not sought.

## Consent

Written informed consent for publication of the study data was obtained from all participants and/or from the parents or legal guardians of minor participants prior to enrollment. Participants (or their legal representatives) consented to the use of their information for research purposes and for publication. No individually identifiable patient information is included in this manuscript.

## Conflicts of Interest

The authors declare no conflicts of interest.

## Supporting information




**Supporting File 1**: puh270272‐sup‐0001‐SuppMat.docx

## Data Availability

The data supporting the findings of this study contain sensitive human‑ and household‑level information and are therefore not publicly available. De‑identified data may be made available from the corresponding author upon reasonable request, subject to ethical and confidentiality considerations.
