# Supplementary material for: Intestinal Parasites in a Rural Highland Tourist Community of Nepal: Diversity, Prevalence, and Associated Factors in Humans and Livestock
Source: Public Health Chall. 2026 May 11;5(2):e70272. doi: 10.1002/puh2.70272 (PMC13159712; doi:10.1002/puh2.70272)
Supplement: Supplementary file 1 — Supporting File 1: puh270272‐sup‐0001‐SuppMat.docx [file PUH2-5-e70272-s001.docx]

**Supplementary File S1: Questionnaires for Sociodemographic, contextual lifestyle & behavioral factors**

**Section A: Sociodemographic Information**

1. Age (in years): __________Sex: -M…. F-- NOID:
2. Number of family members living in your home: __________
3. Can you read and write?
   ☐ Yes ☐ No
4. Occupation:
   ☐ Agriculture ☐ Other
5. Source of drinking water:
   ☐ Tap/Other ☐ Well
6. Type of house:
   ☐ Concrete ☐ Mud/Other
7. Toilet facility:
   ☐ Yes ☐ No (Open field defecation)
8. Have you had diarrhea in the past 7 days?
   ☐ Yes ☐ No

**Section B: Hygiene & Lifestyle**

1. Do you use soap when washing your hands?
   ☐ Yes ☐ No
2. Do you trim your nails regularly (weekly)?
   ☐ Yes ☐ No
3. Do you wash vegetables and fruits before eating?
   ☐ Yes ☐ No
4. Do you walk barefoot outdoors?
   ☐ Yes ☐ No
5. Are you vegetarian?
   ☐ Yes ☐ No
6. Do you eat raw meat?
   ☐ Yes ☐ No
7. Have you dewormed within the past 3 months?
   ☐ Yes ☐ No

**Section C: Animal-Related Information**

1. Status of shed:
   ☐ Captive ☐ Free-range ☐ Both
2. Type of animal:
   ☐ Adult ☐ Calf
3. Anti-helminthic drugs given:
   ☐ Yes ☐ No
4. Gender of animal:
   ☐ Male ☐ Female
